# Computational Insights into the Molecular Mechanisms of *Coptis chinensis* Franch. in Treating Chronic Atrophic Gastritis: An Integrated Network Pharmacology, Machine Learning, and Molecular Dynamics Study

**DOI:** 10.3390/ijms262411998

**Published:** 2025-12-12

**Authors:** Chengxiang Hu, Yang Liu, Yiyao Ding, Yue Jin, Weiwei Han

**Affiliations:** School of Life Sciences, Jilin University, Changchun 130012, China; hucx23@mails.jlu.edu.cn (C.H.); liuyangyang@jlu.edu.cn (Y.L.); yiyao25@mails.jlu.edu.cn (Y.D.)

**Keywords:** *Coptis chinensis* Franch., chronic atrophic gastritis, SRC kinase, network pharmacology, molecular dynamics, machine learning

## Abstract

Chronic atrophic gastritis (CAG) is a precancerous gastric condition with limited therapeutic interventions, and the mechanisms underlying the benefits of *Coptis chinensis* Franch. (CCF) remain insufficiently defined. This study employed an integrated computational strategy to clarify the molecular basis of CCF activity against CAG. Network pharmacology was used to identify potential targets of the major CCF constituents berberine, coptisine, and palmatine, followed by molecular docking, machine learning-based IC_50_ prediction, and molecular dynamics simulations. Fifty-eight overlapping targets between CCF compounds and CAG-related genes were identified, highlighting SRC, STAT3, MAPK1, and NFKB1 as central nodes enriched in inflammatory and immune pathways, including TNF and MAPK signaling. Docking analyses revealed strong interactions between all three compounds and SRC kinase, and machine learning models predicted IC_50_ values in the low micromolar range (1.38–1.82 μM). Molecular dynamics simulations further suggest that berberine may stabilize the crucial regulatory regions of SRC, specifically the activation loop. It is hypothesized that this stabilization maintains the inactive conformation of the kinase domain and potentially shields Tyr416 from phosphorylation, thus potentially influencing kinase activation. These findings suggest that CCF may modulate key inflammatory and immune pathways implicated in CAG progression, with SRC emerging as a central node for further investigation.

## 1. Introduction

Chronic atrophic gastritis (CAG) is a progressive, multifactorial disease of the gastric mucosa characterized by chronic inflammation, glandular atrophy, and intestinal metaplasia. It is widely acknowledged as a precancerous lesion that significantly increases the risk of gastric cancer, which remains one of the leading causes of cancer-related mortality worldwide [1]. The global incidence of CAG has shown a rising trend, especially in East Asian countries, where *Helicobacter pylori* infection is prevalent and dietary habits often involve high salt intake, smoked foods, and insufficient consumption of fresh fruits and vegetables [2]. Beyond bacterial infection, autoimmune mechanisms, genetic predispositions, and environmental stressors also contribute to disease progression. Clinically, patients often present with nonspecific symptoms such as epigastric discomfort, bloating, or indigestion, making early diagnosis challenging. The pathological evolution of CAG, progressing from inflammation to atrophy and eventually intestinal metaplasia or dysplasia, underscores the urgent need for effective therapeutic interventions capable of halting or reversing disease progression [3].

Despite decades of research, therapeutic options for CAG remain limited. Conventional treatments primarily target *H. pylori* eradication, acid suppression with proton pump inhibitors [4], or the administration of mucosal protective agents. While these strategies alleviate symptoms and reduce bacterial burden, they do not fundamentally reverse mucosal atrophy or prevent progression to gastric cancer. Moreover, antibiotic resistance has emerged as a major obstacle in *H. pylori* eradication [5], necessitating the exploration of alternative or complementary approaches. In this context, traditional Chinese medicine (TCM) has drawn increasing attention for its multi-component and multi-target characteristics, which may offer therapeutic benefits beyond the limitations of conventional single-target drugs.

*Coptis chinensis* Franch. (CCF), a widely used TCM herb, has been historically prescribed for gastrointestinal disorders, including gastritis, diarrhea, and peptic ulcers. Modern pharmacological studies have revealed that its major alkaloids—berberine, coptisine, and palmatine—exhibit a broad spectrum of biological activities, such as anti-inflammatory, antimicrobial, antioxidant, and anticancer effects. berberine, for instance, has been reported to suppress NF-κB activation, inhibit pro-inflammatory cytokine release, and regulate gut microbiota composition [6]. Coptisine and palmatine, although less extensively studied than berberine, also display promising pharmacological properties [7], including modulation of signaling pathways such as MAPK, PI3K/AKT, and JAK/STAT. These mechanistic insights suggest that CCF alkaloids could exert therapeutic effects on CAG by modulating inflammation, immunity, and epithelial homeostasis. However, the precise molecular mechanisms remain incompletely understood, and systematic computational studies integrating network pharmacology [8] and structural dynamics are scarce.

While traditional approaches have identified individual targets, the systemic mechanism of CCF requires a holistic investigation. In this study, we employed an integrated computational strategy combining network pharmacology to map compound–target interactions, machine learning to predict quantitative bioactivity (IC_50_) [9], and molecular dynamics (MD) simulations to elucidate atomic-level conformational changes. This multi-dimensional framework allows for a comprehensive validation of the therapeutic hypothesis.

Importantly, the selection of representative compounds in this study was based on authoritative pharmacopoeial standards. According to the Chinese Pharmacopoeia (2020 edition), berberine, coptisine, and palmatine are recognized as the major alkaloid constituents of CCF, and their quantification serves as the primary quality control indicator for this medicinal herb. Therefore, these three alkaloids were chosen as the focus of mechanistic exploration, ensuring both pharmacological relevance and consistency with clinical quality standards.

Given these considerations, we hypothesize that CCF alkaloids exert therapeutic effects against CAG by targeting SRC and related signaling pathways. To test this hypothesis, we developed an integrated computational strategy combining network pharmacology, molecular docking, machine learning-based IC_50_ prediction, and molecular dynamics simulations. Specifically, we aimed to (1) identify overlapping targets of CCF alkaloids and CAG-related genes; (2) determine core signaling pathways enriched by these targets; (3) evaluate the binding affinities and interaction modes of berberine, coptisine, and palmatine with key targets; (4) predict inhibitory potency against SRC using machine learning models; and (5) explore the dynamic effects of ligand binding on SRC conformational stability, with a focus on the linker region and activation loop (A-loop).

By integrating these complementary approaches, this study seeks to provide comprehensive mechanistic insights into the multi-target, multi-pathway actions of CCF in CAG treatment. The findings not only advance our understanding of the molecular pharmacology of this traditional medicine but also highlight the utility of combining network pharmacology, machine learning, and MD simulations as a generalizable strategy for natural product research.

## 2. Results

### 2.1. Prediction Results of CCF Active Component Targets

To systematically predict the potential targets of the main active components of CCF, SwissTargetPrediction and SuperPred were used. In SwissTargetPrediction, the top 100 predicted targets for each compound were retained. In SuperPred, “Known strong binders” entries were preserved, and from “Additionally predicted targets” those with prediction probability greater than 50% were selected. Targets from the two methods were combined and duplicates removed, yielding a total of 311 putative targets related to CCF active components.

For chronic atrophic gastritis (CAG)-related disease targets, DisGeNET and GeneCards were queried with the keyword “chronic atrophic gastritis”. From DisGeNET, targets with disease association score ≥ 0.01 were retained; from GeneCards, genes with Category = “Protein coding” and Relevance score > 1 were kept. DisGeNET returned 203 CAG-related genes and GeneCards returned 734; after merging and deduplication, 808 CAG-related targets were obtained.

The intersection between the 311 CCF-related targets and the 808 CAG-related targets was computed and visualized by a Venn diagram (Figure S1), showing 58 overlapping targets. These overlapping targets were taken forward for PPI network construction and subsequent analyses.

### 2.2. PPI Network Analysis and Hub Target Screening

The 58 intersecting targets were uploaded to the STRING database to construct a protein–protein interaction (PPI) network (species set to Homo sapiens, minimum interaction score (0.4) as described in Section 4.3, Network Type set to full string), and isolated nodes were removed. The exported data were imported into Cytoscape v3.10.1 for network visualization and topological parameter analysis.

**Figure 1:**
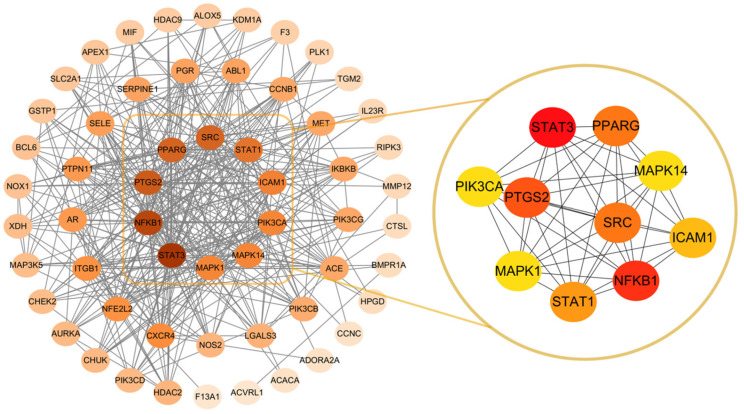
PPI network (**left**): Constructed from the STRING database and visualized by Cytoscape v3.10.2. The depth of the node color indicates the size of the Degree value (the darker the color indicates the number of node connections), and the edge represents the interaction between proteins. Core target subnetwork (**right**): The top 10 key targets screened based on the Degree algorithm using Cytoscape’s CytoHubba plugin.

The resulting PPI network comprised 58 nodes and 523 edges, with a relatively high average node degree, indicating dense interconnections among the overlapping targets. Using the CytoHubba plugin and ranking by degree and other centrality measures (degree, betweenness, etc.), the top 10 hub targets were identified as STAT3, SRC, NFKB1, PTGS2, MAPK1, MAPK14, PIK3CA, STAT1, ICAM1, and PPARG. These core targets occupy central regulatory positions within the network and were selected for subsequent docking and further study. The PPI network (left) and the extracted core-target subnetwork (right) are shown in Figure 1.

### 2.3. GO and KEGG Enrichment Analysis of Intersecting Targets

GO and KEGG enrichment analyses were performed on the 58 intersecting targets. GO enrichment (BP, CC, MF) indicated that these targets were mainly involved in inflammatory responses, immune regulation, cell signal transduction, and angiogenesis-related biological processes. The most significant BP terms included “positive regulation of cytokine production”, “response to lipopolysaccharide”, and “response to bacterial-derived molecules”, suggesting that the targets may modulate cytokine-mediated inflammation and antibacterial immune responses, which are relevant to CAG pathogenesis. CC enrichment highlighted membrane-associated structures such as “membrane raft” and “membrane microdomain”, while MF enrichment emphasized “protein serine/threonine kinase activity” and “DNA-binding transcription factor binding”.

**Figure 2:**
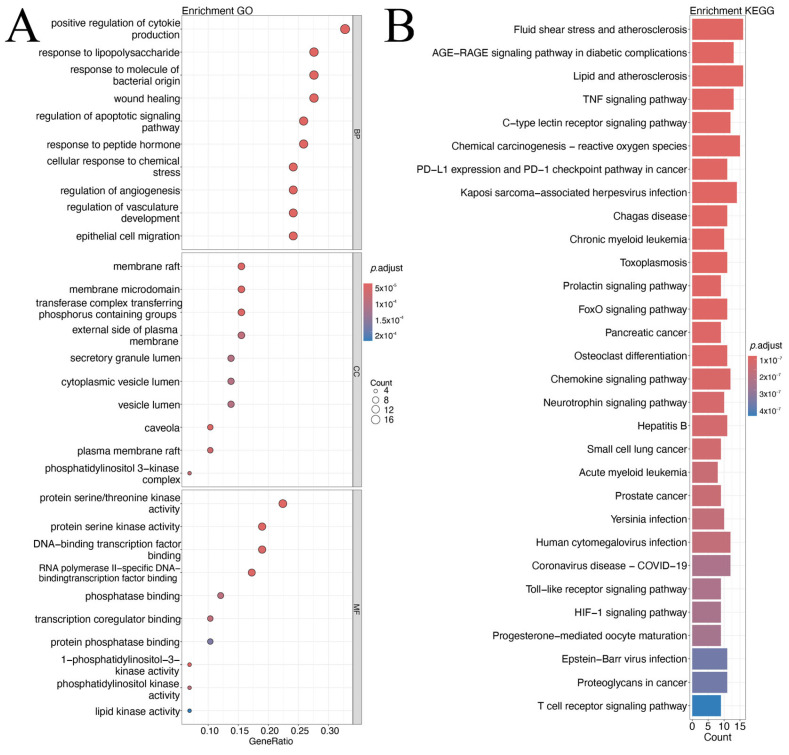
Intersection targets GO (**A**) and KEGG (**B**) Enrichment Analysis Results. Bubble plots show significant concentrations in biological processes (BP), cellular components (CC), and molecular functions (MF). The x-axis shows the proportion of genes (GeneRatio), the bubble size represents the gene count (Count), and the color depth reflects the adjusted *p* value (*p*. adjust, *p*. adjust < 0.05 is Significant). The bar graph shows significantly enriched KEGG pathways, with pathway names on the y-axis, gene counts on the x-axis (Count), and color depth indicating adjusted *p* values (*p*. adjust, *p*. adjust < 0.05 is significant).

KEGG pathway analysis identified multiple pathways associated with inflammation, oxidative stress, infection and cancer. Top enriched pathways included those related to atherosclerosis and shear stress, AGE-RAGE signaling, lipid and atherosclerosis, TNF signaling, and chemokine signaling. Cancer-related pathways (e.g., PD-1/PD-L1 checkpoint, pancreatic cancer, prostate cancer) and infection-related pathways were also enriched. Overall, the enrichment results may offer some support for the possibility that the overlapping targets could be implicated in inflammation suppression, pathogen response, and tissue repair—mechanistic themes that appear to align, to a certain extent, with potential CCF effects on CAG (Figure 2A,B).

### 2.4. Molecular Docking of Core Targets

Molecular docking was performed for the ten selected core targets (MAPK1, MAPK14, PIK3CA, ICAM1, STAT1, SRC, PPARG, PTGS2, NFKB1 and STAT3) against the three CCF alkaloids. PDB IDs and docking box centers used for each target are provided in Table 1. Docking was conducted with AutoDock Vina v1.1.2 using a semi-flexible protocol (rigid protein, flexible ligand).

Docking scores (binding energies, kcal·mol^−1^) showed generally favorable binding of the three alkaloids to most targets. Notably, SRC demonstrated the lowest (most favorable) docking scores among all targets: berberine: −10.1 kcal/mol; coptisine: −10.8 kcal/mol; palmatine: −9.3 kcal/mol. This suggests that SRC possesses a highly favorable binding pocket geometry for the three alkaloids, which is confirmed by a low docking score (interpreted here as a semi-quantitative estimation of binding potential rather than a definitive measure of inhibitory potency), indicating that SRC is a high-affinity target approaching the positive control (the Quinazoline inhibitor obtained a score of −10.2 kcal/mol). Other targets such as MAPK1 (berberine: −8.4, coptisine: −9.6, palmatine: −8.0) and MAPK14 (−8.9, −8.7, −8.3) also showed good affinities. Conversely, PPARG and PTGS2 displayed relatively higher (less favorable) docking scores (PPARG: −2.0 to −3.7; PTGS2: 0.9 to 4.8), potentially reflecting incompatible binding pockets or hydrophilicity at those sites. ICAM1, STAT1, NFKB1 and STAT3 had intermediate docking scores (approximately −5.5 to −7.1). A heatmap of docking scores across the three alkaloids and ten targets is shown in Figure S2.

Although STAT3, MAPK1, and NFKB1 were also identified as top-degree hub targets in the PPI network, SRC was prioritized for in-depth structural and dynamic analysis for three strategic reasons: (1) Upstream Regulation: SRC is a non-receptor tyrosine kinase that acts as a critical upstream regulator, physically phosphorylating and activating both STAT3 and MAPK signaling pathways. Targeting SRC thus offers the potential to simultaneously modulate multiple downstream inflammatory cascades enriched in our KEGG analysis. (2) Binding Affinity: In our preliminary docking screening, SRC consistently exhibited the lowest binding energy (strongest affinity) with all three alkaloids compared to other hub targets (see Docking Results). (3) Structural Applicability: The kinase domain of SRC has high-quality crystal structures available with well-defined regulatory regions (activation loop), making it an ideal candidate for reliable atomistic MD simulations to investigate conformational stability mechanisms.

**Figure 3:**
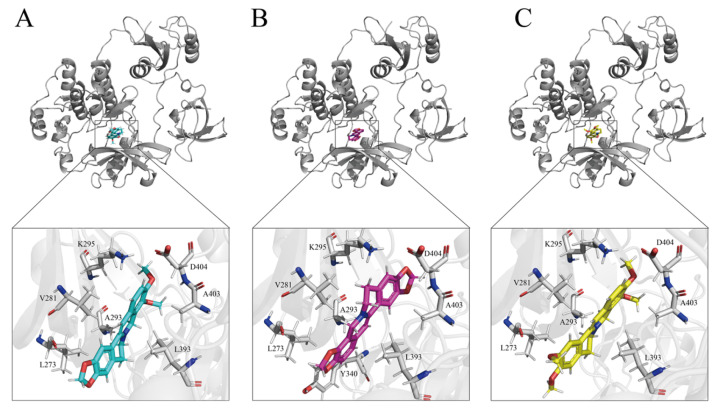
Molecular docking analysis results of berberine (**A**), coptisine (**B**), palmatine (**C**) and Src protein in active components of *Coptis chinensis* Franch.

Detailed docking-mode analyses of the three alkaloids with SRC revealed common interactions: berberine, coptisine and palmatine all positioned in the ATP-binding pocket and formed hydrogen bonds with residues such as K295, D404, and A403, supplemented by hydrophobic contacts with V281, A293, L273, L393 and, in some cases, Y340. coptisine displayed the strongest average affinity and, in SRC complexes, made additional contacts (e.g., with Y340) that may enhance stability. Representative 3D binding poses and interaction annotations are shown in Figure 3.

### 2.5. Machine Learning Prediction of Small-Molecule IC_50_ Against SRC

To quantitatively predict the inhibitory potency of the three alkaloids against SRC, bioactivity data for SRC-related compounds were retrieved from ChEMBL. An initial dataset of 3915 compounds with experimental IC_50_ and SMILES was obtained. After quality control (removal of missing values, duplicates, and obvious outliers), 2306 compounds remained for model training. The histogram of data distribution shows a good fit with the normal fitting curve (μ = 7.25, σ = 2.15), and the R^2^ value is 0.9854 (Figure S3). This indicates that the sample data distribution conforms to the normal distribution assumption, which is conducive to constructing an accurate prediction model.

RDKit was used to compute hybrid descriptors (combining Extended-Connectivity Fingerprints and RDKit descriptors) as molecular descriptors. IC_50_ values were converted to pIC_50_ (pIC_50_ = −log_10_(IC_50_ × 10^−9^)). Low-variance feature filtering (variance < 0.15) was applied to reduce noise.

A comparison of the chemical space revealed that the average Tanimoto similarity between the training set (ChEMBL) and the external test set (BindingDB) was approximately 0.1384, suggesting that the model successfully generalized to compounds outside the core structural clusters of the training data. And to ensure the reliability of the predictions, a domain of applicability analysis was performed. We calculated the maximum Tanimoto similarity (Morgan fingerprints) between the three alkaloids and the ChEMBL training set. berberine, coptisine, and palmatine exhibited maximum similarity scores of 0.2597, 0.2414 and 0.2295, respectively, which were significantly higher than the similarity of the training set and external test set. These values indicate that the alkaloids fall well within the structural chemical space of the model, justifying the reliability of the predicted pIC_50_ values.

Four regression models were trained and optimized via Optuna: XGBRegressor, GradientBoosting, Random Forest and CatBoost. Model performance yielded R^2^ values in the range 0.744–0.795 and MSE values in the range 0.448–0.560. Specifically, GradientBoosting achieved R^2^ = 0.795 and MSE = 0.448, while XGBRegressor had R^2^ = 0.791 and MSE = 0.459; CatBoost gave R^2^ = 0.789 and MSE = 0.463. Cross-validation fold scores varied (mean CV score shown in Figure S6), with an average CV score of 0.54 (max 0.67 at fold 8, min 0.45 at fold 1), indicating reasonable model stability.

**Figure 4:**
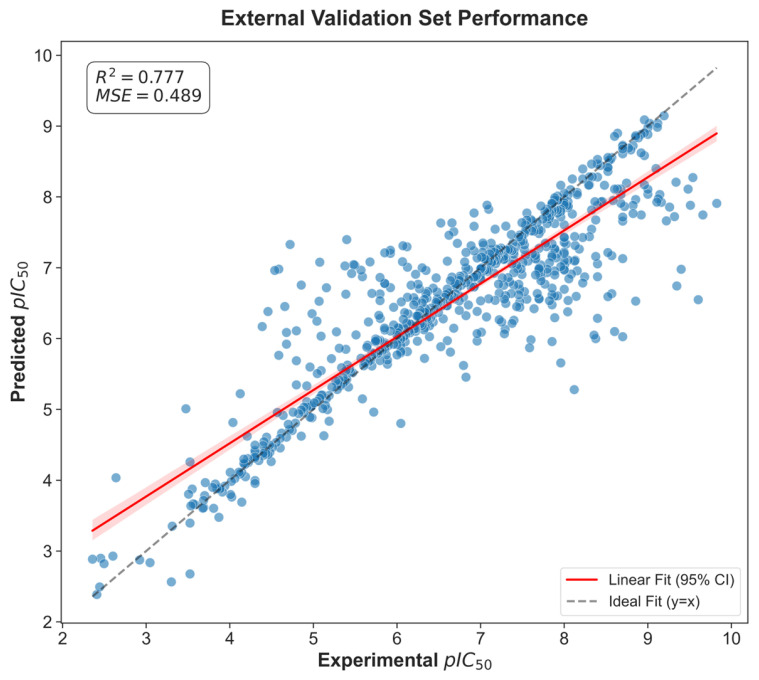
Performance of the model on an external test set.

The GradientBoosting model employed the following hyperparameters: ‘n_estimators’: 500, ‘learning_rate’: 0.07, ‘max_depth’: 4, ‘subsample’: 0.81, ‘min_samples_split’: 8. To assess the robustness of the models, predictions were evaluated on the external BindingDB dataset (n = 721). Model performance on this independent dataset was lower than that obtained by cross-validation on the ChEMBL training set, as expected, but still demonstrated acceptable predictive power (R^2^ = 0.777, MSE = 0.489) (see Figure 4). These results confirm that the model retained the ability to generalize to structurally diverse compounds beyond the training distribution.

**Figure 5:**
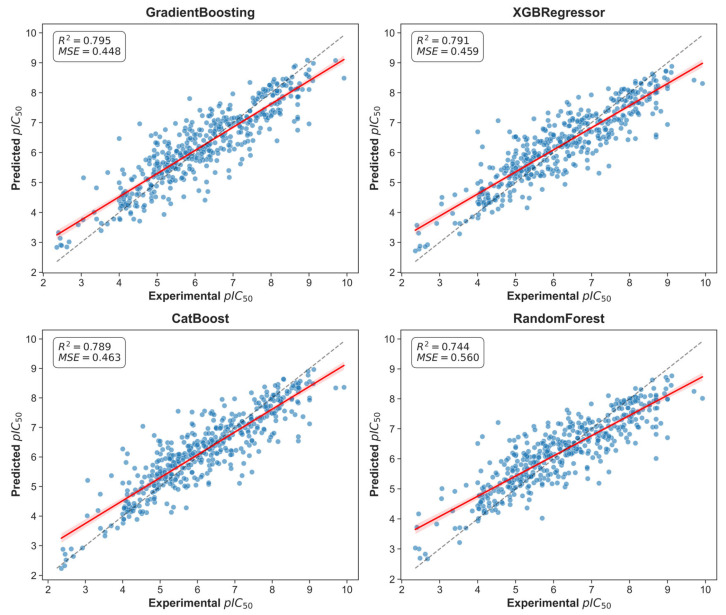
Scatter plot of predicted true values and predicted values of Src protein IC_50_.

Using the optimal model (GradientBoosting), predicted pIC_50_ values for berberine, coptisine and palmatine were 5.86, 5.74 and 5.77, respectively, which correspond to IC_50_ estimates of ≈1.38 μM, 1.82 μM and 1.70 μM. Thus, berberine exhibited the highest predicted inhibitory activity (IC_50_ ≈ 1.38 μM), followed by palmatine and coptisine. These prediction results were validated by plotting predicted vs. experimental values (Figure 5), residuals (Figure S4), error distributions (Figure S5) and cross-validation scores (Figure S6). It is important to state that the cross-validated performance of our machine-learning model (R^2^ ≈ 0.78, MSE ≈ 0.48) corresponds to an approximate prediction uncertainty of ±0.7 pIC_50_ units (based on the MSE), which is typical for kinase bioactivity datasets. Therefore, the predicted activities should be interpreted as qualitative relative trends rather than precise quantitative potency estimates.

Feature importance analysis using the SHAP framework revealed that the model relies on a combination of macroscopic physicochemical properties and specific structural fragments to predict bioactivity (Figure S7).

Among the top 15 most influential features, SlogP_VSA10, SMR_VSA9, and BCUT2D_LOGPHI emerged as the leading determinants. SlogP_VSA10 and BCUT2D_LOGPHI are descriptors associated with hydrophobicity and lipophilicity, indicating that the hydrophobic interactions within the SRC binding pocket are critical for ligand potency. SMR_VSA9 (related to molar refractivity) and MinPartialCharge suggest that polarizability and electrostatic distribution also play significant roles in binding affinity.

Additionally, specific Morgan fingerprint bits, such as FP_1452, FP_1586, and FP_491, were identified as key structural motifs, likely corresponding to specific chemical substructures essential for interacting with the kinase domain. This hybrid reliance on global physicochemical descriptors (e.g., SlogP, SMR) and local substructures (fingerprint bits) confirms that the model captures robust structure–activity relationships.

### 2.6. Protein Structural Stability Analysis from MD Simulations

To examine the dynamic behavior and stability of SRC in complex with the three alkaloids, MD simulations were performed and analyzed via RMSD, Rg, SASA and RMSF metrics.

**Figure 6:**
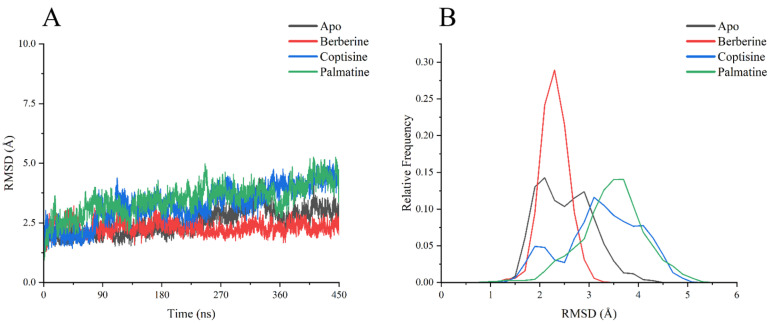
RMSD analysis chart of each composite system (**A**) RMSD value change curve with time, (**B**) RMSD relative frequency distribution data statistics.

RMSD analysis of backbone Cα atoms over the 0–450 ns production runs (Figure 6A) indicated that all systems reached relative equilibrium by approximately 90 ns. The Apo and berberine complexes showed the smallest RMSD fluctuations and lowest mean RMSD, stabilizing mainly within ~2.0–2.5 Å, suggesting high structural stability and close retention of the initial conformation. The coptisine complex exhibited moderately higher RMSD (~3.0–3.5 Å) and broader fluctuations, implying modest conformational changes. The palmatine complex displayed the largest RMSD values and most pronounced fluctuations, particularly after ~300 ns where RMSD rose and sustained at ~4.0–5.0 Å, indicating substantial conformational rearrangement or relaxation.

RMSD frequency distributions (Figure 6B) corroborated these observations: berberine produced the sharpest peak near 2.0 Å (most concentrated), Apo had a wider peak around 2.0 Å, coptisine’s distribution peaked near 3.0 Å, and palmatine’s distribution shifted toward 4.0 Å with the broadest spread.

**Figure 7:**
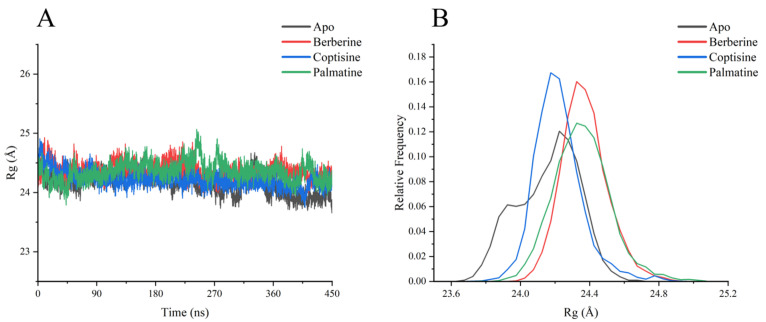
Rg analysis diagram of each system (**A**) Rg value change curve with time, (**B**) Rg relative frequency Rate distribution data statistics.

Radius of gyration (Rg) traces (Figure 7) showed all systems fluctuated around ~24 Å, indicating overall retention of a compact globular structure. Apo and berberine had the smallest Rg fluctuations (major peaks ~24.0–24.5 Å), while coptisine and palmatine displayed slightly higher mean Rg values and broader fluctuation ranges; palmatine in particular showed a transient increase to ~25.0 Å around 270–300 ns, suggesting a slight loosening of compactness.

**Figure 8:**
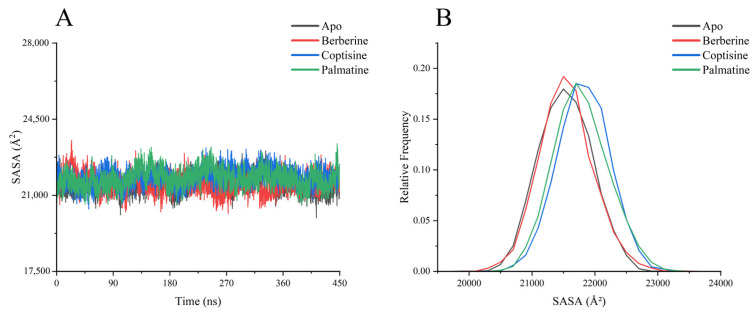
SASA analysis chart of each system (**A**) SASA value change curve with time, (**B**) SASA Relative frequency distribution data statistics.

SASA analyses (Figure 8) revealed overall SASA values concentrated around ~21,000 Å^2^, with minor differences among systems. berberine complex showed the smallest SASA fluctuations and slightly lower average SASA, consistent with its stabilizing effect on global structure.

**Figure 9:**
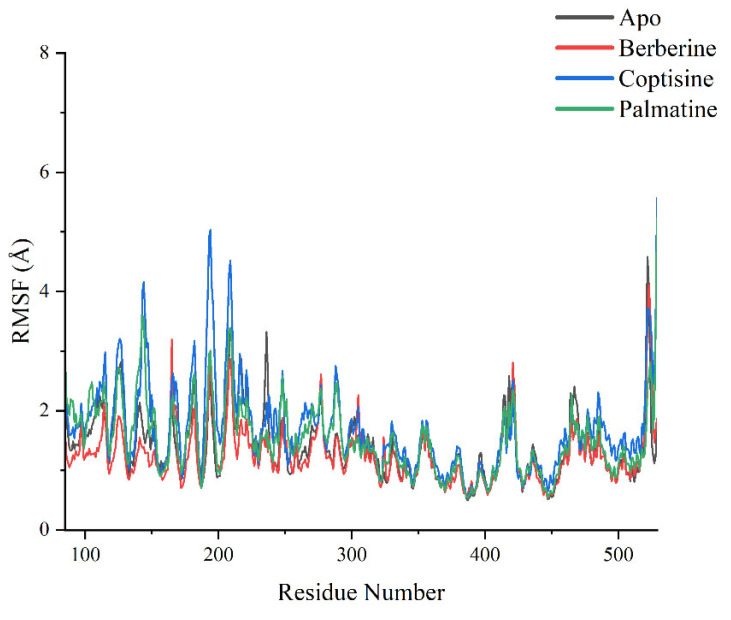
RMSF analysis diagram of each system.

RMSF profiles (Figure 9) identified several regions with high residue flexibility across systems, including residues 100–150, 190–220, 300–350 and 500–550, typically corresponding to loop regions or termini. Notably, residues around 190–220 and ~520 exhibited substantially higher RMSF in coptisine and palmatine complexes compared to Apo and berberine. For example, near residue ~200, coptisine yielded RMSF peaks approaching ~5.0 Å while Apo and berberine were below ~3.0 Å; near residue ~520, palmatine’s RMSF approached ~6.0 Å, much higher than other systems. These local flexibility increases in coptisine and palmatine complexes likely contribute to their higher global RMSD values.

Of particular mechanistic interest, the 230–240 region—the linker region connecting SH3/SH2 domains to the kinase domain and implicated in Src autoinhibition—showed decreased flexibility upon ligand binding. The Apo system presented higher RMSF in this region, whereas berberine, coptisine and palmatine complexes reduced RMSF, suggesting ligand-induced stabilization that may favor autoinhibited conformations.

MM-PBSA analysis (see Table 2) revealed that coptisine exhibited the strongest gas-phase interactions (ΔG_gas = −10.52 kcal/mol), but also the largest solvation penalty, resulting in an overall ΔG_total of 0.19 kcal/mol. berberine showed the most favorable binding free energy (ΔG_total = −3.23 kcal/mol), whereas palmatine yielded the weakest binding (1.41 kcal/mol). These results indicate that berberine is the thermodynamically preferred ligand among the three compounds.

### 2.7. Secondary Structure Dynamics (DSSP)

DSSP-based secondary structure analysis focused on two key regions: the 230–240 linker region and the 404–418 activation loop (A-loop).

**Figure 10:**
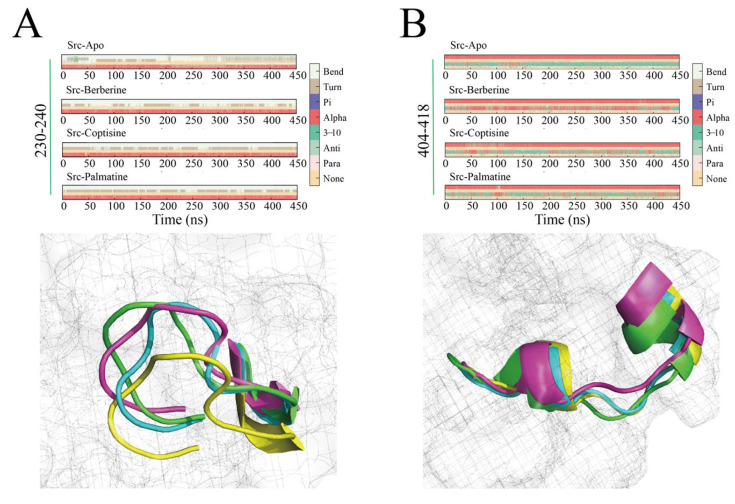
Secondary structure changes of Src kinase during simulation, with berberine (light blue), coptisine(pink), palmatine (yellow). Sequence 230–240 (**A**), 404–418 (**B**).

For residues 230–240 (Figure 10A), the Apo system predominantly displayed coil/none and frequent transitions among turn and bend states, indicating high flexibility. In contrast, the berberine complex primarily maintained turn and bend conformations throughout the 450 ns simulation, with substantially fewer transitions to coil states—indicating stabilization. coptisine and palmatine complexes showed similar stabilization trends relative to Apo, though with occasional transient formations of 3_10_ or α-helical elements—palmatine exhibiting sporadic short 3_10_/α-helix appearances in later simulation stages.

For the 404–418 activation loop (Figure 10B), which includes the critical Tyr416 residue, the Apo system began with an α-helix that progressively unwound after ~100 ns into turn/bend/coil conformations—potentially exposing Tyr416 and enabling phosphorylation. By contrast, the berberine complex preserved a nearly continuous α-helical conformation in residues 404–418 through most of the 450 ns run, indicating sustained stabilization of the A-loop and likely masking of Tyr416 from phosphorylation. coptisine and palmatine also promoted α-helical propensity in this region but with less persistence and occasional helix unwinding relative to berberine. Collectively, DSSP findings indicate that berberine most effectively stabilizes both the linker region and activation loop. This suggests that ligand binding may influence A-loop flexibility and secondary-structure preference locally, which may modulate kinase regulatory behavior, thereby potentially reinforcing Src’s autoinhibited state and preventing activation via Tyr416 phosphorylation; coptisine and palmatine exert similar but comparatively weaker stabilizing effects.

## 3. Discussion

Chronic atrophic gastritis (CAG) is a well-recognized precancerous lesion of gastric cancer, and its pathological progression is closely associated with long-term inflammation, immune dysregulation, and structural remodeling of gastric mucosa. Therefore, understanding the molecular mechanisms underlying potential therapeutic strategies for CAG is of great clinical importance. In traditional Chinese medicine (TCM), CCF has been extensively used to treat gastrointestinal disorders, and its alkaloid constituents—berberine, coptisine, and palmatine—are the main bioactive components recorded in the Chinese Pharmacopoeia. Although numerous pharmacological studies have confirmed their anti-inflammatory, antibacterial, and antitumor activities, the precise molecular mechanisms by which these compounds act against CAG remain insufficiently elucidated.

In this study, by integrating network pharmacology, molecular docking, machine learning-based activity prediction, and molecular dynamics (MD) simulations, we systematically explored the mechanism of CCF in CAG treatment. Network analysis revealed 58 overlapping targets between CCF compounds and CAG-related genes, with SRC, STAT3, MAPK1, and NFKB1 identified as hub nodes. These findings are consistent with existing reports suggesting that chronic gastric inflammation involves aberrant activation of pro-inflammatory signaling cascades, particularly the MAPK and JAK/STAT pathways, which contribute to epithelial damage and progression to intestinal metaplasia [10]. Thus, the enrichment of these pathways among CCF targets strongly suggests that CCF may exert therapeutic effects through the regulation of inflammatory signaling and immune responses.

Molecular docking and machine learning predictions provided further insights into the compound–target interactions. All three alkaloids displayed favorable docking affinities toward SRC. Berberine was predicted to exhibit the strongest inhibitory activity (IC_50_ ≈ 1.38 μM), while both coptisine and palmatine also showed good binding affinities and promising inhibitory effects. This result is intriguing, as berberine has traditionally been considered the most pharmacologically active constituent of CCF. It suggests that coptisine and palmatine, despite their relatively lower abundance, may play a more significant role in the inhibition of specific kinase targets such as SRC. This highlights the importance of considering multiple alkaloids in the therapeutic evaluation of CCF, as synergy among constituents may underlie its clinical efficacy [11]. Moreover, the docking analysis showed that all three alkaloids form stable hydrogen bonds and hydrophobic interactions with residues within the ATP-binding pocket of SRC, consistent with known mechanisms of small-molecule kinase inhibition.

The machine learning models predicted IC50 values in the low micromolar range (1.38–1.82 μM) for the three alkaloids. While sub-micromolar potency is often desired in early drug discovery, these predicted values are relevant in the context of *Coptis chinensis* pharmacology. Since CCF is administered orally for the treatment of gastric conditions like CAG, the local concentration of alkaloids in the gastric lumen and mucosa may reach levels significantly higher than systemic plasma concentrations. This high local bioavailability suggests that even moderate-affinity compounds can exert effective biological activity at the target tissue site, partially mitigating concerns regarding the generally low systemic bioavailability of protoberberine alkaloids.

The MD simulation results further validated the stability of compound–SRC complexes, while also providing dynamic structural insights. Particularly noteworthy was the observation that berberine binding induced stabilization of the SRC activation loop (residues 404–418) and seems to maintain an α-helical structure that potentially masks Tyr416 from phosphorylation. This structural rearrangement is highly significant because Tyr416 phosphorylation is critical for full activation of SRC kinase. It is hypothesized that by stabilizing the inactive conformation, berberine may prevent aberrant SRC activation and thereby suppress downstream inflammatory signaling.

Interestingly, coptisine and palmatine exhibited somewhat weaker stabilizing effects, which may correspond to their higher RMSD values and increased flexibility in specific regions. Thus, our findings provide structural hypotheses that require future validation using full-length SRC or experimental assays.

Our comparative analysis highlights distinct roles for the three alkaloids. berberine consistently exhibited relatively more favorable binding free energy and the strongest capacity to stabilize the SRC activation loop, positioning it as the primary pharmacophore for kinase inhibition. In contrast, coptisine and palmatine, while structurally similar, showed slightly higher conformational flexibility in MD simulations. This suggests they may serve as complementary agents, potentially targeting different conformational states or working synergistically to enhance the overall therapeutic efficacy of the crude herbal extract.

It is important to note that the MD simulations were performed using a single 450 ns trajectory for each system due to computational constraints. While the simulations reached equilibrium, the lack of replicate trajectories limits the statistical confidence of the observed conformational changes. Furthermore, the binding free energies calculated via MM-PBSA should be interpreted qualitatively, as energy differences smaller than 3 kcal/mol typically fall within the method’s error margin. Regarding secondary structure, DSSP analysis quantitatively indicated that berberine maintained a significantly higher α-helical content in the activation loop (85%) compared to the Apo state (7%) throughout the simulation stability plateau.

These mechanistic findings align with the broader pharmacological roles of SRC in inflammation and cancer. SRC kinase is known to regulate multiple downstream pathways, including MAPK, JAK/STAT, and PI3K-Akt [12], all of which were significantly enriched in our KEGG analysis. Persistent activation of these pathways has been implicated not only in CAG progression but also in gastric carcinogenesis. Therefore, inhibition of SRC by CCF alkaloids may represent a key molecular mechanism linking TCM practice with modern biomedical understanding.

Beyond CAG, our results may also have implications for other gastrointestinal and inflammatory diseases. For example, berberine has been reported to ameliorate ulcerative colitis and metabolic syndrome through modulation of similar signaling pathways. The convergence of evidence suggests that CCF and its alkaloids may act as broad-spectrum regulators of inflammatory signaling [6,7]. Furthermore, our machine learning approach highlights the utility of computational prediction in natural product research, providing quantitative insights into compound potency that complement traditional docking analyses.

Despite these promising findings, the limitations of the integrated computational approach must be acknowledged. Network pharmacology predictions rely on databases such as SwissTargetPrediction and DisGeNET, which are constructed based on probabilistic associations, chemical similarity, and text mining. Consequently, these data sources may inherently contain noise, false positives, and chemical similarity biases, lacking direct mechanistic validation. To mitigate these limitations, we employed a multi-tier screening strategy incorporating molecular docking, machine learning, and molecular dynamics to cross-validate the initial network predictions. However, the reported interactions remain theoretical hypotheses that require rigorous experimental verification.

In conclusion, this study provides a computational framework suggesting that CCF may exert therapeutic effects on CAG through multi-target and multi-pathway interactions, with SRC kinase emerging as a central node based on network and docking analyses. Among the alkaloids, berberine showed the most pronounced computationally predicted structural influence on the isolated SRC kinase domain, including a tendency to stabilize local secondary-structure elements within the activation loop region. Overall, our integrated network pharmacology, machine learning, and molecular dynamics approach generates mechanistic predictions that can guide future experimental research. Subsequent biochemical and cellular studies will be essential to validate these computational findings and to further define the therapeutic potential of CCF in the prevention and treatment of CAG.

## 4. Materials and Methods

### 4.1. Active Compound Selection

**Figure 11:**
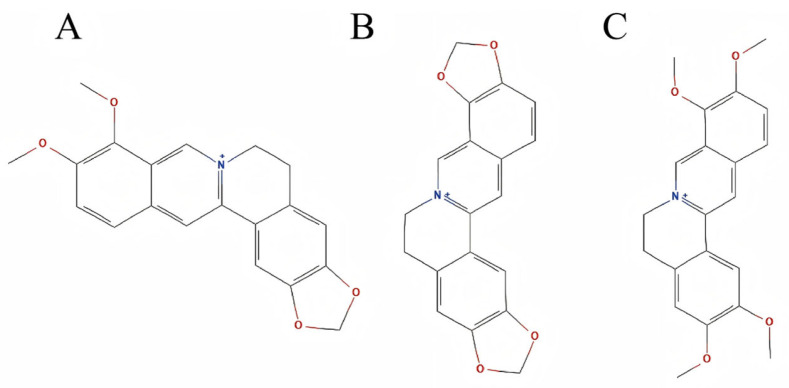
The chemical structures of berberine (**A**), coptisine (**B**), and palmatine (**C**).

According to the Chinese Pharmacopoeia (2020 edition), berberine, coptisine, and palmatine are recorded as the major alkaloid constituents of CCF, and their content is routinely used as the primary quality control index for this medicinal herb. These three alkaloids account for the pharmacologically dominant components of CCF and are considered reliable markers for evaluating both its authenticity and therapeutic potential. The chemical structures of the three alkaloids are presented in Figure 11. In addition to their standardized use in pharmacopoeial testing, modern pharmacological studies have consistently reported their anti-inflammatory, antimicrobial, and anticancer effects, which are relevant to the pathophysiology of chronic atrophic gastritis (CAG). On this basis, berberine, coptisine, and palmatine were selected as representative active compounds for subsequent computational analyses in this study.

### 4.2. Potential Target Prediction

The potential targets of active components in CCF were predicted using two widely applied online platforms: SwissTargetPrediction (http://www.swisstargetprediction.ch/ (accessed on 13 October 2024)) [13] and SuperPred 3.0 (https://prediction.charite.de/index.php (accessed on 8 October 2024)) [14]. On the SwissTargetPrediction platform, the top 100 predicted targets for each compound were retained to ensure high-confidence targets. On the SuperPred 3.0 platform, all entries labeled as “Known strong binders” were kept, and from the “Additionally predicted targets” category only those with a prediction probability greater than 50% were selected. Through the combined use of these two platforms, a comprehensive set of putative protein targets for the CCF active components was obtained.

To collect disease-related targets associated with chronic atrophic gastritis (CAG), we queried DisGeNET (https://www.disgenet.org/ (accessed on 21 October 2024)) [15,16] and GeneCards (https://www.genecards.org/ (accessed on 21 October 2024)) [17] using the keyword “chronic atrophic gastritis.” From DisGeNET we filtered by disease association score (score ≥ 0.01). From GeneCards we retained genes with Category = “Protein coding” and Relevance score > 1. The intersection of predicted compound targets and disease-associated genes was then computed and visualized with a Venn diagram for subsequent analysis.

### 4.3. Protein–Protein Interaction (PPI) Network Construction

The intersecting targets were submitted to the STRING database v12.0 (http://string-db.org/ (accessed on 18 December 2024)) [18] to construct a protein–protein interaction (PPI) network. The species was set to Homo sapiens, and non-interacting (isolated) nodes were removed. The interaction data exported from STRING (TSV format) were imported into Cytoscape v3.10.1 [19] for visualization and topological analysis. Node importance in the network was ranked using the CytoHubba plugin v0.1 [20], and key hub targets were identified for further study.

### 4.4. GO and KEGG Enrichment Analysis

Based on the core target information, Gene Ontology (GO) [21] and Kyoto Encyclopedia of Genes and Genomes (KEGG) pathway enrichment analyses [22,23] were performed using R v4.4.1 [24] and related Bioconductor packages (including BiocManager v1.30.25, clusterProfiler v4.12.6, AnnotationHub v3.12.0, org.Hs.eg.Db v3.19.1, pathview v1.44.0, dplyr v1.1.4 and ggplot2 v3.5.2). GO enrichment focused on Biological Process (BP), Cellular Component (CC), and Molecular Function (MF). KEGG enrichment was used to identify relevant signaling pathways. Significance thresholds were set at *p* < 0.01 and q < 0.01. Enrichment results were visualized using bar plots and bubble charts for clear presentation.

### 4.5. Molecular Docking of Key Targets

Molecular docking was carried out for selected key target proteins using AutoDock Vina v1.1.2 [25,26]. Protein crystal structures were obtained from the Protein Data Bank (PDB; http://www.rcsb.org/pdb (accessed on 30 December 2024)) [27,28]. Prior to docking, protein structures were prepared by removing water molecules and any co-crystallized ligands, and by adding necessary hydrogen atoms and charge assignments. The docking grid box was defined according to the position of ligands in the crystal structures, taking the co-crystallized ligand binding site as the active pocket center.

Ligand structures were retrieved from the PubChem database (https://pubchem.ncbi.nlm.nih.gov/ (accessed on 31 December 2024)) [29,30] and subjected to energy minimization using Discovery Studio 2021; the minimized structures were saved in PDBQT format for docking. A semi-flexible docking protocol was employed in which the protein was kept rigid, and the ligands were allowed conformational flexibility within the binding pocket. Docking outputs were ranked by binding energy; the conformation with the lowest binding energy (highest affinity) was selected as the initial structure for subsequent molecular dynamics simulations.

To improve the robustness of molecular docking results, each ligand was docked multiple times using AutoDock Vina with repeated random seeds to increase sampling diversity. For each target–ligand pair, Vina generated several candidate binding poses, and the lowest-energy pose that consistently appeared across repeated runs was selected as the final representative conformation. This strategy ensures that the chosen binding mode is not a stochastic outlier but a reproducible low-energy configuration.

Docking results and interaction modes were visualized with PyMOL (Version 3.0 Schrödinger, LLC., Portland, OR, USA) [31,32,33] and AutoDock Vina’s visualization utilities. Key interactions such as hydrogen bonds, hydrophobic contacts, and π–π stacking were annotated to illustrate ligand–protein binding patterns.

Finally, we emphasize that Vina scores represent semi-quantitative estimations of binding affinity. Therefore, docking was used primarily to suggest the plausibility of ligand–receptor interactions rather than to infer precise potency differences among compounds.

### 4.6. Machine Learning Prediction of Small-Molecule IC_50_

To predict the half-maximal inhibitory concentration (IC_50_) of the three natural small molecules (berberine, coptisine and palmatine) against Src kinase, bioactivity data associated with Src were retrieved from the ChEMBL database (https://www.ebi.ac.uk/chembl/ (accessed on 20 May 2025)) [34,35]. Compounds in ChEMBL with experimentally measured IC_50_ values and available SMILES strings [36] were collected. Data preprocessing included removal of missing values, duplicate molecules and obvious outliers to ensure dataset quality. The ChEMBL dataset was randomly divided into a training set and a validation set with an 80% and 20% split, respectively.

Molecular representations were computed using RDKit (version: 2024.03.6) [37]; specifically, Hybrid descriptors, which combine Extended-Connectivity Fingerprints (ECFP/Morgan fingerprints) [38] and RDKit physicochemical descriptors [37]. Morgan fingerprints were calculated following the algorithms described by Morgan and Rogers & Hahn, with a radius of 2 and 2048-bit vector length. RDKit 2D descriptors included constitutional, topological, and physicochemical parameters computed by RDKit’s descriptor calculators. IC_50_ values were transformed to pIC_50_ by pIC_50_ = −log_10_(IC_50_ × 10^−9^). Feature selection included low-variance filtering to remove features with near-zero variance.

Four regression algorithms were employed for model building: XGBRegressor [39], GradientBoosting [40], Random Forest [41] and CatBoost [42]. Hyperparameter optimization for each model was performed using Optuna v4.3.0 [43] with a Bayesian optimization strategy. Model performance was evaluated by ten-fold cross-validation, using coefficient of determination (R^2^) and mean squared error (MSE) as the primary metrics.

To further evaluate the generalization performance of the machine-learning models, an external test set was constructed using bioactivity data retrieved from the BindingDB database (https://www.bindingdb.org/rwd/bind/index.jsp (accessed on 23 November 2025)). A total of 792 SRC-related inhibitors with experimentally measured IC_50_ values and available SMILES strings were downloaded. After removing duplicates, non-standardized structures, and compounds lacking valid measurements, 721 unique molecules remained and were used as an independent external test set.

The chemical spaces of the ChEMBL training set and the BindingDB external test set were compared by calculating the average Tanimoto similarity based on Morgan fingerprints. The results confirmed that while the two datasets exhibit overlap in descriptor space, the external set maintains sufficient chemical diversity to serve as an independent validation benchmark.

All IC_50_ values were converted to pIC_50_ (pIC_50_ = −log_10_(IC_50_ × 10^−9^)). No molecules in the external test set overlapped with the ChEMBL training set, ensuring full independence between training and evaluation datasets.

After selecting the optimal model, the SMILES strings of berberine, coptisine, and palmatine were input to obtain predicted pIC_50_ values and corresponding IC_50_ estimates.

To enhance model interpretability and identify the key molecular features driving bioactivity predictions, we employed the SHapley Additive exPlanations (SHAP) framework. The SHAP values were calculated for the optimal GradientBoosting model to quantify the contribution of each feature—including both Morgan fingerprint bits and RDKit physicochemical descriptors—to the predicted pIC_50_ values. This analysis allows for the prioritization of features that most significantly influence SRC kinase inhibition.

### 4.7. Molecular Dynamics Simulations

Molecular dynamics (MD) simulations were carried out using AMBER 22 [44]’s PMEMD.CUDA module [45] with GPU acceleration. Protein atoms were parameterized with the ff14SB force field [46], while ligand parameters were generated by Antechamber based on the General AMBER Force Field (GAFF) [47]. Ligand atomic charges were derived using the RESP fitting method to better represent electrostatic distributions.

Each protein–ligand complex was solvated in a cubic water box with a margin of 15 Å, employing the TIP3P water model [48]. Systems were neutralized by adding Na^+^ counterions [49] as required.

Prior to production runs, systems underwent energy minimization to remove steric clashes: a two-stage minimization was performed, first by steepest descent for 500 steps and then by conjugate gradient for 500 steps, totaling 1000 minimization steps. Temperature equilibration proceeded under NVT conditions, gradually heating the system from 0 K to 300 K over 50 ps with a time step of 0.002 ps (i.e., 25,000 steps). SHAKE constraints [50] were applied to all bonds involving hydrogen atoms. Temperature control was implemented with a Langevin thermostat using a collision frequency of 2.0 ps^−1^.

Following heating, density equilibration was performed for 50 ps and pressure equilibration under NPT conditions for 500 ps, using the Berendsen barostat for pressure control. Long-range electrostatics were treated with the Particle Mesh Ewald (PME) method [51], and a non-bonded cutoff of 8 Å was used.

Production MD simulations were conducted for 450 ns with a 2 fs time step under periodic boundary conditions. Trajectory snapshots were saved every 2 ps. Resulting trajectories were analyzed with CPPTRAJ v6.18.1 [52] for structural stability (RMSD), residue flexibility (RMSF), radius of gyration (Rg), solvent-accessible surface area (SASA), and other dynamic properties. Representative conformations were visualized with PyMOL and VMD v1.9.4a53.

The solvation free energy (Δ*G_solv_*) was calculated as the sum of the polar (Δ*G_polar_*) and nonpolar (Δ*G_npolar_*) contributions. The polar term was calculated using the Poisson–Boltzmann (PB) model. The nonpolar solvation energy (Δ*G_npolar_*) was calculated based on the solvent-accessible surface area (SASA) using the linear relationship: Δ*G_npolar_* = *γ·SASA* + *β*, where standard constants were applied. Specifically, the nonpolar contribution consists of a cavity term (linearly related to SASA) and a van der Waals dispersion term (*E_disper_*).

Due to computational constraints, a single 450 ns production run was performed for each system. Future studies involving replicate trajectories are needed to ensure statistical sampling and robustness.

### 4.8. Secondary Structure Analysis

Protein secondary structure assignments were computed from MD trajectories using the secstruct [53] command in CPPTRAJ, which calls the DSSP program to generate per-residue secondary structure annotations over time. DSSP codes (0–7) were mapped to secondary structure types as follows: 0 = coil/none, 1 = β-strand/extended, 2 = single β-bridge, 3 = 3_10_-helix, 4 = α-helix, 5 = π-helix, 6 = turn, and 7 = bend.

DSSP output was post-processed to focus on the relative proportions and temporal evolution of major secondary structure elements—α-helix, β-sheet (including parallel and antiparallel strands), and random coil. Plotting optimizations included aligning the time axis to the 450 ns production length, color-coding secondary structure categories (α-helix in warm tones, β-sheet in cool tones, coil in neutral tones), and adjusting plot framing, line widths and legends for clarity. Time-resolved secondary structure maps were plotted using Gnuplot v6.0 and exported in vector format for high-quality figure presentation.

## 5. Conclusions

This study systematically investigated the therapeutic mechanisms of *Coptis chinensis* Franch. (CCF) against chronic atrophic gastritis (CAG) by integrating network pharmacology, molecular docking, machine learning-based activity prediction, and molecular dynamics simulations. The results revealed that CCF exerts its effects through multi-component and multi-target interactions, with SRC, STAT3, MAPK1, and NFKB1 identified as hub targets. Enrichment analyses highlighted the MAPK, JAK/STAT, and PI3K-Akt signaling pathways as critical nodes in the regulation of inflammation and immune responses.

Among the three representative alkaloids, berberine exhibited the strongest predicted inhibitory activity toward SRC, and seems to uniquely stabilized the activation loop of SRC, computationally predicted to prevent its phosphorylation and subsequent activation. These findings not only provide mechanistic insights into the anti-CAG effects of CCF but also suggest that different alkaloids may act synergistically to modulate key inflammatory signaling pathways.

Overall, our work generates mechanistic predictions that can guide future experimental research to validate these molecular hypotheses, while also demonstrating the value of combining network pharmacology with machine learning and molecular dynamics to elucidate the molecular basis of traditional medicines. Future biochemical and cellular experimental validation will be essential to confirm these preliminary computational findings and further clarify the therapeutic potential of CCF.

## Figures and Tables

**Table 1:** The molecular docking parameters for targets.

Targets	PDB ID	Box Site	Box Size
MAPK1	6GDQ	x = 6.99 y = 8.35 z = 44.95	x = 19.912, y = 12.013, z = 15.085
MAPK14	6SFO	x = 0.05 y = 1.07 z = −19.15	x = 15.423, y = 11.935, z = 20.774
PIK3CA	7V9R	x = −23.81 y = 9.35 z = 27.61	x = 25.597, y = 15.403, z = 13.277
ICAM1	5MZA	x = −0.11 y = −18.35 z = 9.78	x = 20.000, y = 20.000, z = 20.000
STAT1	1YVL	x = −18.20 y = 2.68 z = 104.80	x = 20.000, y = 20.000, z = 20.000
SRC	2H8H	x = 20.67 y = 20.03 z = 57.69	x = 17.985, y = 15.228, z = 11.533
PPARG	8B8W	x = −0.25 y = 19.62 z = 38.26	x = 15.678, y = 19.621, z = 38.263
PTGS2	5F1A	x = 41.97 y = 23.97 z = 240.06	x = 9.392, y = 8.269, z = 7.600
NFKB1	2O61	x = 7.92 y = −56.73 z = 4.40	x = 14.215, y = 13.1, z = 15.76
STAT3	6TLC	x = 39.35 y = 20.38 z = 95.99	x = 20.000, y = 20.000, z = 20.000

**Table 2:** Binding free energy MM-PBSA results (kcal/mol).

Energy Type	Src—Berberine	Src—Coptisine	Src—Palmatine
Evdw	−6.34 ± 0.25	−8.30 ± 0.31	−5.74 ± 0.24
Eelec	−1.56 ± 0.09	−2.22 ± 0.10	−1.55 ± 0.09
Epb	4.68 ± 0.20	5.66 ± 0.21	4.30 ± 0.19
Enpolar	0 ± 0	−5.00 ± 0.20	−3.73 ± 0.18
Edisper	0 ± 0	10.05 ± 0.35	8.14 ± 0.32
ΔGgas	−7.91 ± 0.32	−10.52 ± 0.40	−7.30 ± 0.31
ΔGsolv	4.68 ± 0.20	10.72 ± 0.36	8.72 ± 0.32
ΔTotal	−3.23 ± 0.13	0.19 ± 0.08	1.41 ± 0.06

## Data Availability

The original data presented in the study are openly available in zenodo at https://doi.org/10.5281/zenodo.17786564.

## Supplementary Materials

The following supporting information can be downloaded at: https://www.mdpi.com/article/10.3390/ijms262411998/s1.

## Abbreviations

The following abbreviations are used in this manuscript:

CAG	chronic atrophic gastritis
PPI	protein-to-protein interaction
CCF	*Coptis chinensis* Franch.
GO	Gene Ontology
KEGG	Kyoto Encyclopedia of Genes and Genomes
DSSP	Define Secondary Structure of Proteins
MD	Molecular Dynamics
MSE	Mean Squared Error
Rg	Radius of Gyration
RMSD	Root-Mean-Square Deviation
RMSF	Root-Mean-Square Fluctuation
SASA	Solvent-Accessible Surface Area
SHAP	SHapley Additive exPlanations
