# Supplementary material for: Computational Insights into the Molecular Mechanisms of Coptis chinensis Franch. in Treating Chronic Atrophic Gastritis: An Integrated Network Pharmacology, Machine Learning, and Molecular Dynamics Study
Source: Int J Mol Sci. 2025 Dec 12;26(24):11998. doi: 10.3390/ijms262411998 (PMC12732890; doi:10.3390/ijms262411998)
Supplement: Supplementary file 1 [file ijms-26-11998-s001.zip › ijms-4027324-supplementary.pdf]

Supplementary Materials

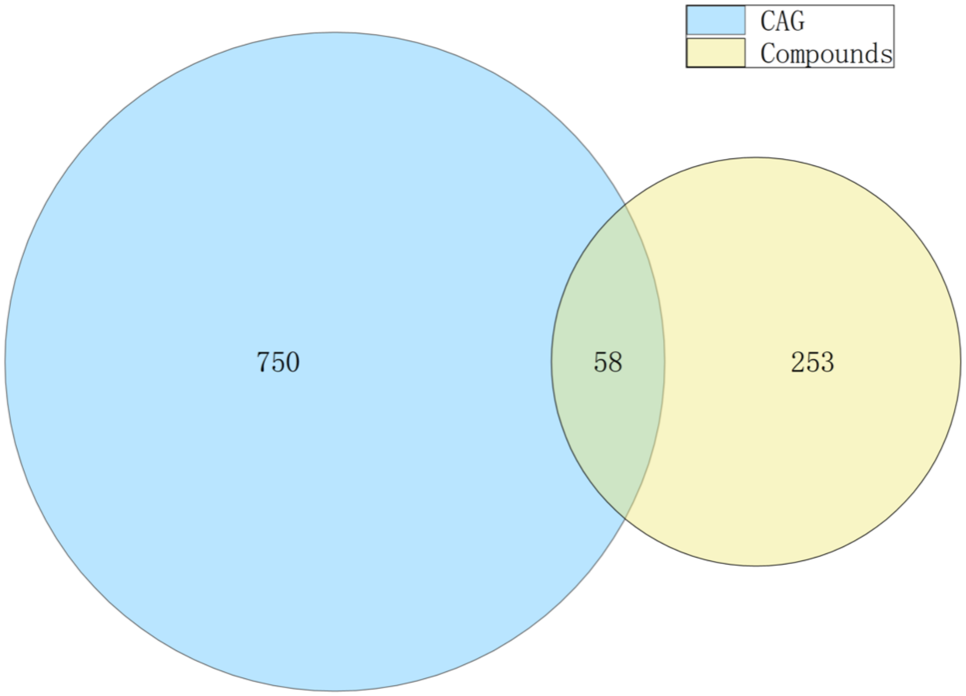

Figure S1. Venn diagram illustrating the intersection of predicted targets. The diagram displays the overlap between the 311 potential targets of CCF active ingredients and the 808 CAG-related disease targets, identifying 58 common targets used for subsequent analysis.

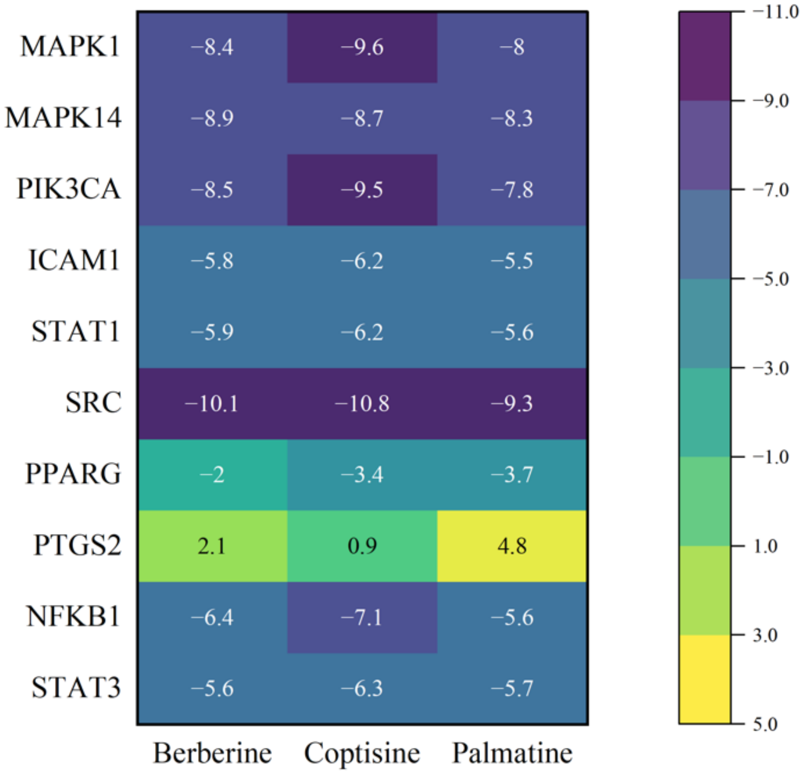

Figure S2. Heatmap of molecular docking scores. The plot visualizes the binding affinities (kcal/mol) between the three alkaloids (berberine, coptisine, palmatine) and the top 10 hub targets.

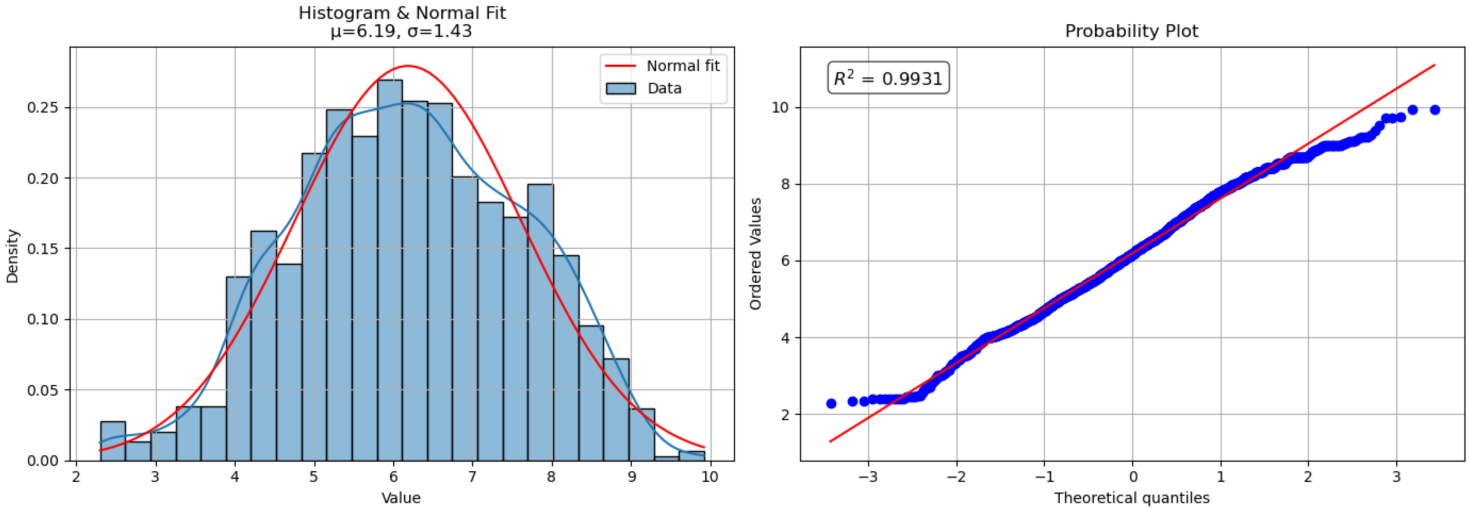

Figure S3. Data distribution of the ChEMBL dataset. The histogram shows the normal distribution fit of pIC_{50} values for the SRC kinase inhibitor dataset used for machine learning model training.

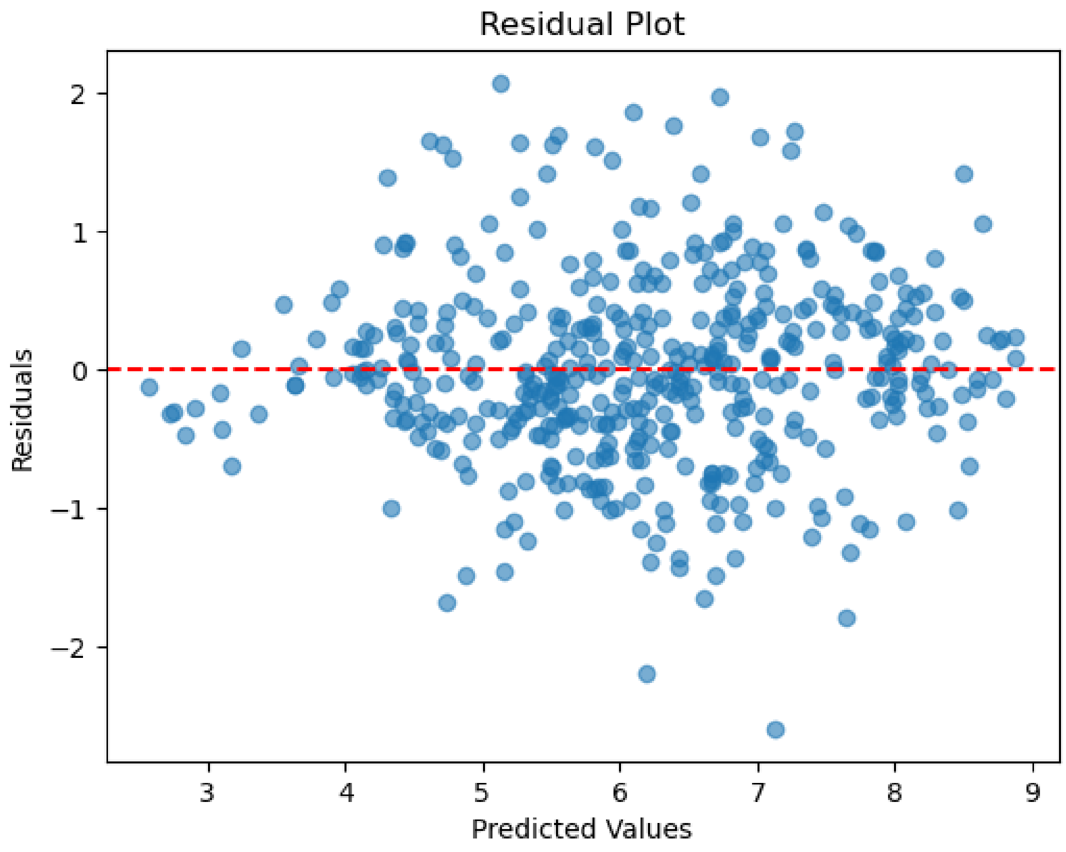

Figure S4. Residue plot for Src protein IC_{50} prediction: The scatter plot shows the residuals change with predicted value, the x-axis is the predicted value, the y-axis is the residual, and the red line is the zero residual line. The residuals are randomly distributed, indicating the consistency of the model predictions.

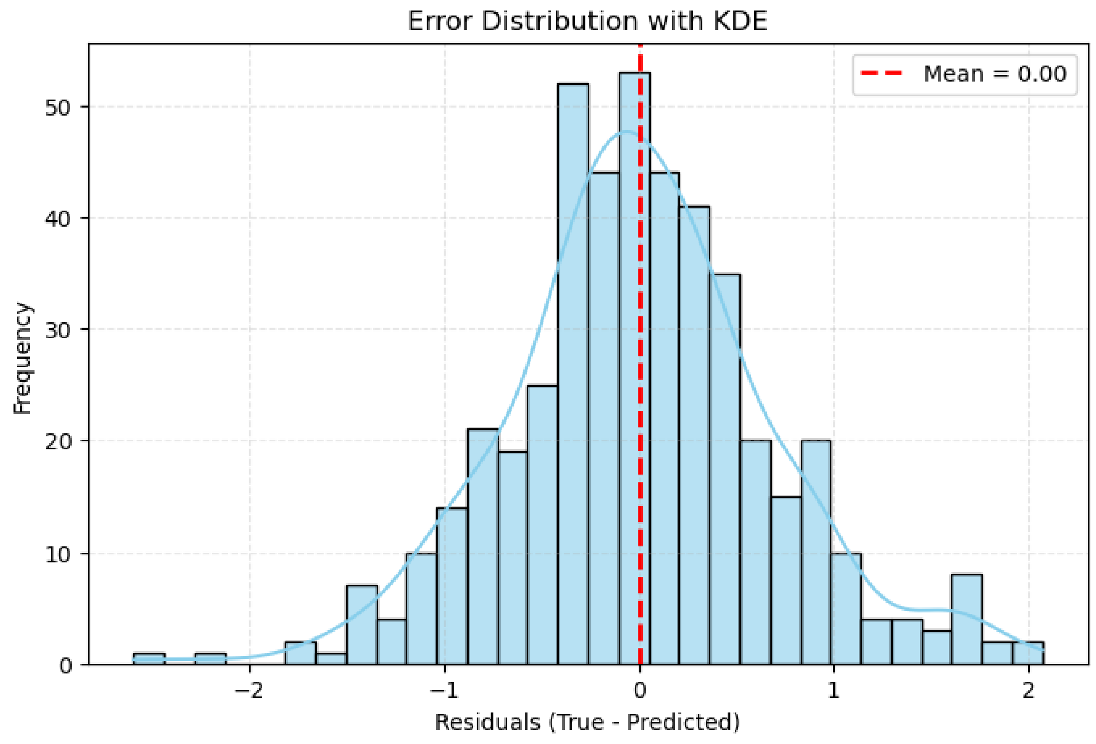

Figure S5. Error distribution histogram. This figure confirms that the prediction errors follow a normal distribution centered near zero.

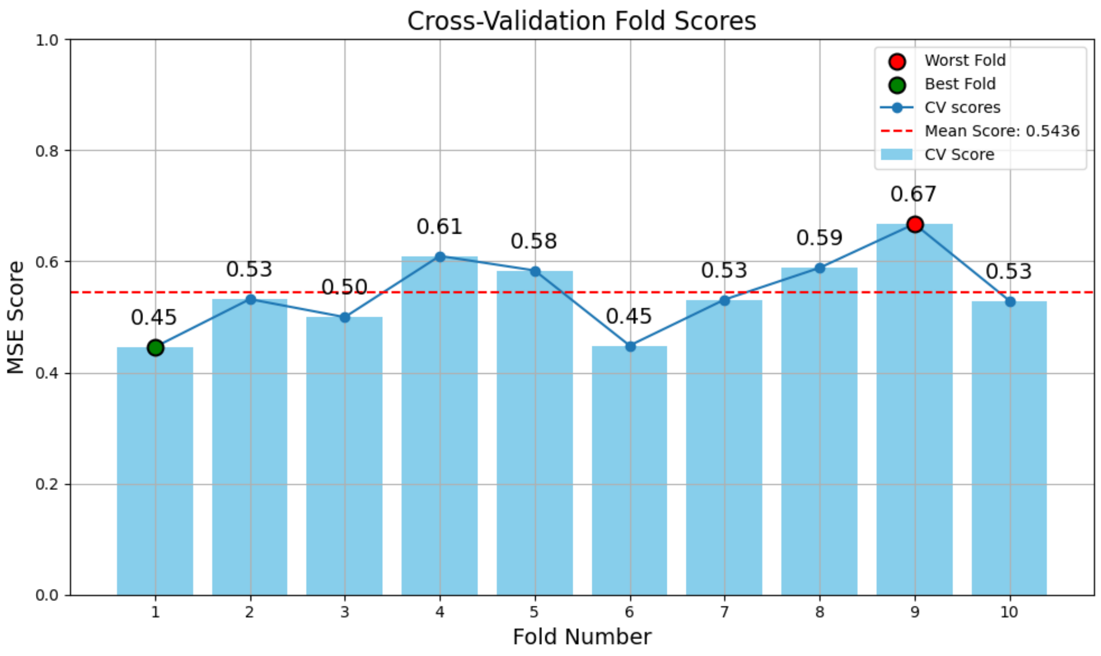

Figure S6. Cross-validation scores.The bar chart shows the MSE scores for each of the 10 folds in the cross-validation process, demonstrating model stability.

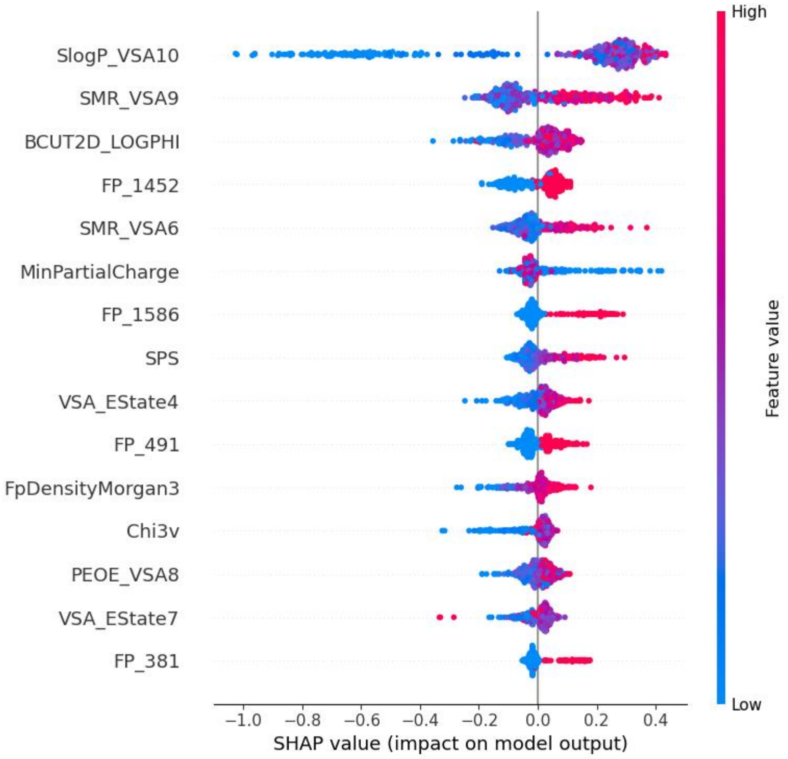

Figure S7. SHAP summary plot of the top 15 features for the GradientBoosting model. The y-axis lists the top 15 most important features ranked by the sum of SHAP value magnitudes, including RDKit physicochemical descriptors (e.g., SlogP_VSA10, SMR_VSA9, BCUT2D_LOGPHI) and Morgan fingerprint bits (e.g., FP_1452). The x-axis represents the SHAP value, indicating the impact on the predicted pIC_{50} (positive values indicate a contribution to higher potency). Each dot represents a single compound, with color denoting the feature value (red = high, blue = low).
